# Conformal Load-Bearing Antenna Structures—Mechanical Loading Considerations

**DOI:** 10.3390/s22010048

**Published:** 2021-12-22

**Authors:** Rowan Healey, Kelvin J. Nicholson, John Wang, Joel E. Patniotis, Taylor Lynch, Wing K. Chiu

**Affiliations:** 1 Department of Mechanical and Aerospace Engineering, Monash University, Clayton, VIC 3800, Australia; tlyn0001@student.monash.edu (T.L.); Wing.Kong.Chiu@monash.edu (W.K.C.); 2 Defence Science and Technology Group, Aerospace Division, Fishermans Bend, VIC 3207, Australia; kelvin.nicholson@dst.defence.gov.au (K.J.N.); John.Wang@dst.defence.gov.au (J.W.); Joel.Patniotis@dst.defence.gov.au (J.E.P.)

**Keywords:** CLAS, HFSS, disbond, radiofrequency

## Abstract

One of the important functions of antennas is to facilitate wireless communication. The IEEE 802.11 is part of the IEEE802 set of local area network technical standards, and specifies the media access control and physical layer protocols for implementing wireless local area network computer communication. The network physical layer protocol with a centre frequency of 2.4 GHz has a bandwidth of 22 MHz. A conformal load-bearing antenna structure (CLAS) facilitating this communication band that is tuned to 2.4 GHz must remain within this bandwidth. The aim of this paper is to investigate the effects of mechanical loading imposed on a load-bearing patch antenna with respect to its ability to remain within the specified bandwidth. The mechanical loading configurations considered include tensile, biaxial, and twisting. This paper will also report on the response of the antenna patch to the presence of a disbond between the metallised antenna and its substrate, which can arise due to fabrication anomalies and operational usage. This numerical work will assist in the design of experimental testing of the mechanical and electromagnetic properties of an embedded CLAS, which will ultimately be used to inform selection of appropriate regions to place patch antennas on load-bearing deformable surfaces.

## 1. Introduction

**Figure 1:**
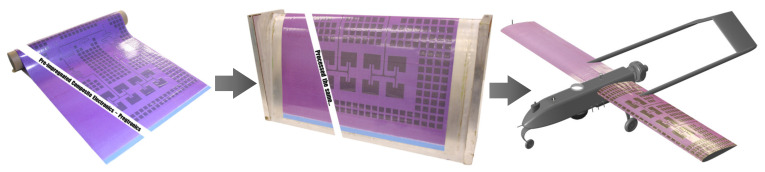
CLAS concept. Prepreg composite material with embedded electromagnetic traces can be cured in aerospace composite structures, such as wing skins on UAV.

Conformal load-bearing antenna structures (CLAS) offer the opportunity to integrate communication and sensing capabilities into load-bearing airframes [1,2,3], as illustrated in Figure 1. Microstrip patch antennas are one simple example that can easily be integrated into typical conformal airframe structures. A significant advantage of this antenna is its ubiquitous use on commercial Uninhabited Aerial Vehicles (UAV). Allowing radiofrequency (RF) devices to be manufactured directly into composite skins would free designers to disperse these systems across the exterior of a platform, greatly increasing the performance of the underlying RF systems by enabling either larger antenna apertures or low frequency operation. In addition, considerable improvements in aerodynamics are achieved through embedding the antenna structures [4,5,6,7]. Conventional antenna structures are prone to damage as a consequence of being exposed externally to the environment. CLAS, being embedded within the platform, are considerably more resilient to such damage [8]. However, CLAS are now subjected to the structural loading of the platform and must be designed to account for this.

To ensure that a CLAS is designed appropriately, it is necessary to understand both its mechanical and electromagnetic behaviour under a variety of loading conditions [9,10,11]. The influence of fatigue loading and damage/failure modes are also of great importance. Through developing an understanding of the conditions under which the CLAS performs ideally and poorly, the optimal integration of CLAS can be ascertained. Investigations have been conducted into the mechanical [12,13,14,15,16] and electromagnetic [17,18,19,20,21,22,23,24] performance of different forms of CLAS.

Yoon et al. performed compressive testing on a CLAS smart skin with a multi-layer sandwich structure, finding the transverse shear moduli of the honeycomb core influenced the buckling load of the smart skin when shear deformation was considerable [12]. Zhong et al. demonstrated a CLAS textile spiral antenna which exhibited no noteworth changes in performance after 300 flexing cycles [13]. Kim et al. investigated how impact damage on the CLAS affected an antenna performance, finding that a relationship between impact damage and antenna performance (as measured by the antenna S11) was observed with the threshold of the impact energy for acceptable antenna function being 1.5 and 1.75J [21]. Conversely, Li et al. demonstrated an inverse method to detect defects in CLAS by assessing the change in the antenna far-field phase response [25].

Fuhong and Shanyi studied the mechanical and electrical performance of a honeycomb sandwich structure under three-point loading, which demonstrated good agreement with FEA [17]. Zhou et al. conducted strain and tensile tests on a conductive nanotube (CNT) sheet as it was bent and stretched, good conductivity, and high flexibility was observed with acceptable performance after 100 degrees of bending and 13% stretching, making it suitable for CLAS applications [19].

Daliri et al. examined the properties of a log-spiral slot antenna through FEA finding that the effect of the slot on strength was substantially lower than other typical slot antennas (like resonant slotted waveguides) while also having the advantage of wideband performance [18]. Further experimental work was done by Daliri et al. to show the compressive strength of a carbon fibre composite plate with a spiral slot is comparable to a plate with a circular hole of similar size and that filling the slots with epoxy resin further enhanced compressive strength [20].

**Figure 2:**
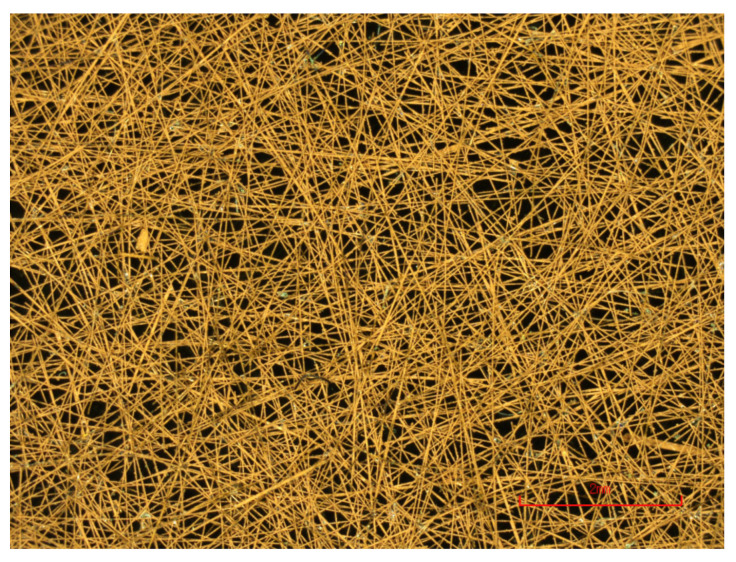
Close-up view of copper-coated carbon veil.

Typically, thin copper sheet is the chosen material for microstrip antenna construction. However, non-woven fiber mats (or surface veils) have the potential to replace copper in a variety of roles, in particular, microstrip antennas (pictured in Figure 1) [26]. Surface veils consist of randomly orientated, short-chopped, carbon fibers and can be metal coated (nickel, copper, etc, see Figure 2), to achieve the desired electromagnetic properties [27]. The increased surface area of the veil compared to copper sheet results in a higher degree of adhesion with the matrix phase; compounded with the increased porosity of the veil, which allows for better resin-to-resin cohesion. The improved adhesion/cohesion of the veil improves the interlaminar fracture toughness, preventing debonding, and damage to the antenna [28]. Therefore, using copper-coated veil for a micro-strip patch antenna would likely improve the longevity and functionality of the CLAS. However, further planned investigation is required to support such claims.

One of the important functions of antennas is to facilitate wireless communication. The IEEE 802.11 is part of the IEEE802 set of local area network technical standards, and specifies the media access control and physical layer protocols for implementing wireless local area network computer communication. The network physical layer protocol with a centre frequency of 2.4 GHz has a bandwidth of 22 MHz [29]. A conformal load-bearing antenna facilitating this communication protocol and is tuned to a center frequency of 2.4 GHz must remain within this bandwidth. The aim of this paper is to investigate the effects of mechanical loading imposed on a CLAS patch antenna with respect to its ability to remain within the specified bandwidth. Given that the resonance of the patch antenna is defined by its geometric parameters, this paper will also endeavour to expound on the effects of the mechanical loading on its quality factor. In this paper, the mechanical loading configurations considered include tensile loading, biaxial loading, and twisting.

The metallised non-woven fiber patch antenna is adhesively bonded to the di-electric substrate. The disbond between the metallised antenna and its substrate can arise due to fabrication anomalies and operational usage. This paper will report on the response of the patch antenna to these types of structural issues. Each scenario was simulated using the ANSYS HFSS software package with the complex S11-parameter being measured. The performance of the antenna will be reported in terms of the shift in the resonant frequency and the quality factor. This numerical work will assist in the design of experimental testing of the mechanical and electromagnetic properties of a veil embedded CLAS. This work will ultimately be used to inform selection of appropriate regions to place patch antennas on load-bearing deformable surfaces, such as the wings of a UAV.

## 2. Materials and Methods

**Figure 3:**
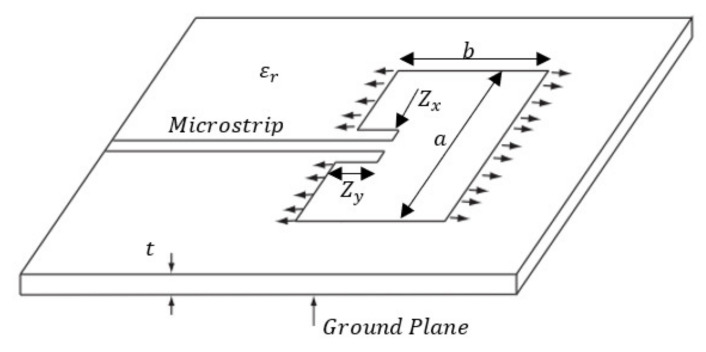
Schematic of a patch antenna.

Figure 3 shows the geometry of a patch antenna. The microstrip feed is inset into the patch antenna by a distance, $Z_{y}$, for impedance matching. The dimension, b, is chosen so that the cavity formed by the conductor on the top plane of the structure is resonant at the designed frequency (2.4 GHz in this case). This causes radiation at the two edges of the antenna as shown.

**Figure 4:**
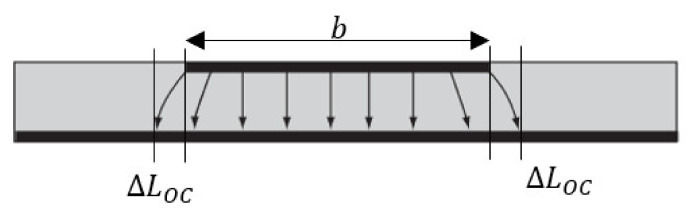
Cross-section of the patch antenna.

The cross-section of the patch antenna is shown in Figure 4. The total length of the cavity is the length of the patch antenna and the effective length at each edge due to the microstrip open-end effect. The fundamental resonant frequency of the patch antenna is defined by the effective length of the patch antenna, $b+2\Delta L_{OC}$ and is expressed as:

(1) $$f_{r}=\frac{c}{2\sqrt{\varepsilon_{re}}\left(b+2\Delta L_{OC}\right)}$$

where c, is the speed of light in vacuum and $\varepsilon_{re}$ is the effective permittivity of the substrate, which is defined in (2), [30].

(2) $$\varepsilon_{re}=\frac{\varepsilon_{r}+1}{2}+\frac{\varepsilon_{r}-1}{2}{\left(1+\frac{10t}{a}\right)}^{-0.5}$$

The effective length of the fringing field is defined as;

(3) $$\frac{\Delta L_{OC}}{t}=0.412\frac{\left(\varepsilon_{re}+0.3\right)\left(\frac{a}{t}+0.264\right)}{\left(\varepsilon_{re}-0.258\right)\left(\frac{a}{t}+0.813\right)}$$

The resonant frequency of the antenna is dependent on the geometry and the material properties of the cavity as shown above. Also implicit is the fact that no change in geometry of the antenna is expected, which is valid if the patch antenna is not integrated in a loading-bearing structure. When using the concept of conformal load-bearing antenna structures (CLAS), the effects of mechanical loading on the antenna resonance is therefore critical. The above equations show that the resonant behaviour of a patch antenna is dependent on effective length given by $b+2\Delta L_{OC}$. Therefore, the patch antenna’s resonance will be affected by its state of deformation when it is subjected to mechanical loading. This is important because the IEEE802.11 Wi-Fi channels has bandwidth of approximately 22 MHz. It is important that the any mechanical loading induced changes in the resonance must not cause the antenna to stray out of band.

The quality factor (Q factor) is another important characteristic of resonant antennas. The Q factor is optimised by matching the impedance of the feedline with patch antenna, which can be achieved by selecting the appropriate $Z_{y}$ and width of the antenna, b. The analyses described in [30] show that this condition is met when $Z_{y}$ is chosen appropriately. It is also noted a tight tolerance of $\frac{Z_{y}}{b}$ must be preserved to maintain a high Q factor, for a given $\frac{a}{b}$ ratio.

The aims of this paper are to report on the effects of mechanical loading on: (i) the resonant behaviour of the CLAS and its potential to stray out of band due to mechanical loading; and (ii) the Q factor of the patch antenna. In the first set of the analyses, the effects of dimensional variation on the Q factor of the patch antenna is first investigated. Following that, the effect of mechanical loading and the presence of disbond due to manufacturing defects on the performance of the CLAS will be discussed. The investigations reported on in this paper will included the following load cases:

- Simple tension;
- Biaxial state of stress;
- Twisting-induced shear loading; and
- Simple tension with disbond on the CLAS arising from manufacturing defect.

**Figure 5:**
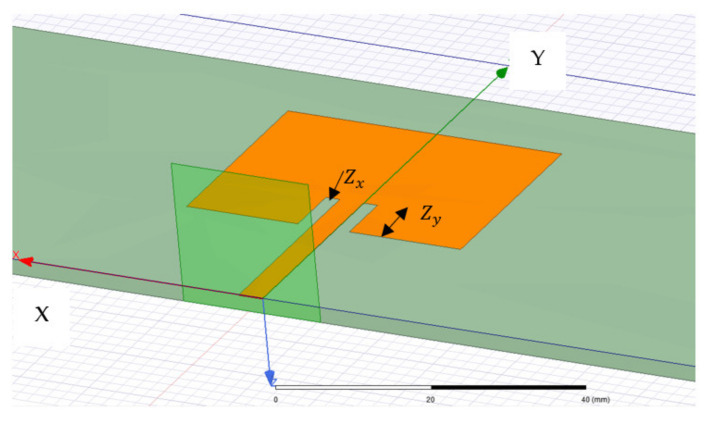
Layout of the embedded patch, GMS EP-280 S-Glass coupon and the waveport used to collect the RF response of the patch.

The coupon and patch models were generated in ANSYS HFSS and then subjected to pre-defined mechanical loading using the Static Structural module in ANSYS. Deformed geometries were then reconstructed in HFSS. The coupons were assumed to have the mechanical properties of GMS EP-280 S-Glass with dimensions of 400 mm × 60 mm × 2.2 mm. The mechanical properties of the substrate are displayed in Table 1 and the mechanical properties of the carbon veil are given in Table 2. The embedded patch (thickness of 0.125 mm) measured 40 mm long, 28.7 mm wide, with a feedline 25.65 mm long and 3.5 mm wide. Inlet gaps 8 mm long and 2 mm wide were utilized to impedance match with the patch being designed to resonant at approximately 2.4 GHz. These dimensions are indicated as “$Z_{x}$, $Z_{y}$” in Figure 4. The electromagnetic properties input into HFSS are displayed in Table 3. A wave-port was placed at the end of the feedline connecting the ground plane (underside of coupon), the dielectric, and the patch, as shown in Figure 5.

For each loading configuration, the effects of the mechanical loading on the resonant behaviour of the patch antenna will be reported. These will be presented in terms of the S11 parameter of the antenna and its quality factor (Q factor).

## 3. Results

### 3.1. Patch Antenna Resonance—The Q Factor

**Figure 6:**
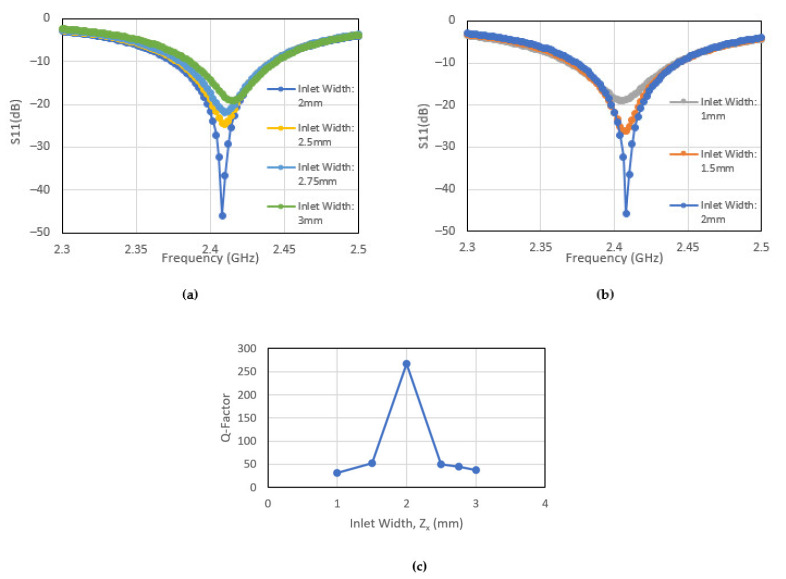
Resonant behavior of patch antenna as a function of changing width of recessed region, $Z_{x}$ (**a**)—increase in $Z_{x}$ from 2 mm; (**b**)—reduction in $Z_{x}$ from 2 mm; and (**c**)—the Q Factor.

**Figure 7:**
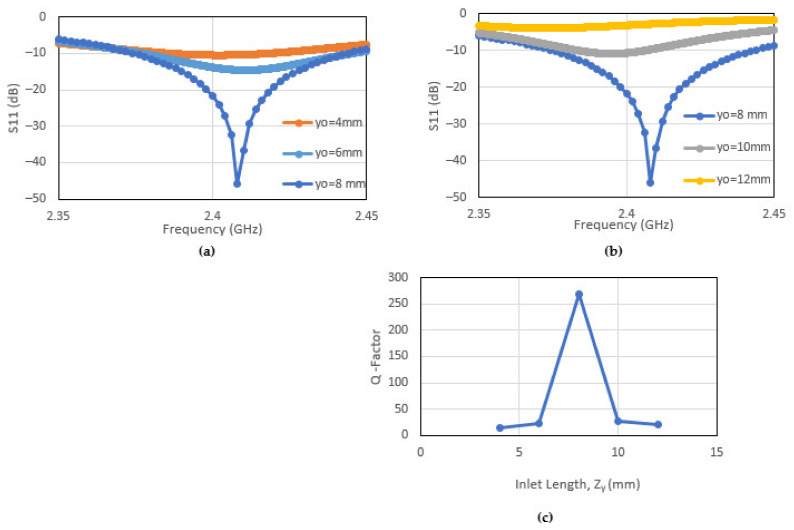
Resonant behavior of patch antenna as a function of changing length of recessed region, $Z_{y}$. (**a**)—reduction in $Z_{y}$ from 8 mm; (**b**)—increase in $Z_{y}$ from 8 mm; and (**c**)—the Q Factor.

The resonance of the patch antenna is dependent on inlet gap dimensions, “$Z_{x}$, $Z_{y}$” as shown in Figure 5. These dimensions are chosen match the impedance between the microstrip line and the patch antenna. The sensitivity of the resonant behaviour and the Q factor will first be investigated. The effects of the change in the dimension $Z_{x}$ whilst keeping $Z_{y}$ fixed, is shown in Figure 6a–c. When the dimension $Z_{y}$ is changed whilst keeping $Z_{x}$ fixed, the corresponding effects are shown in Figure 7a–c. The results show that the dimension of the patch antenna used in this paper is indeed resonant. These results also show that a tight tolerance of the recess dimensions is required to maintain the antenna desired Q factor. More importantly, if the patch antenna is subject to mechanical loading, the corresponding dimensional change will impact on its resonant behaviour. This will be discussed in the following section.

### 3.2. Effects of Uni-Axial Loading

**Figure 8:**
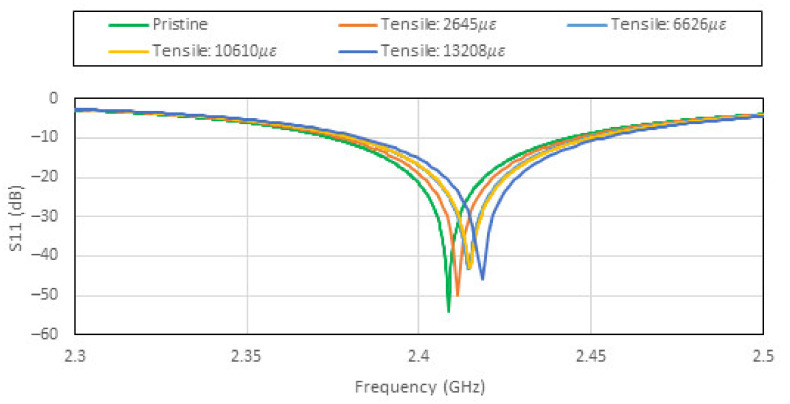
Effects of tensile loading on the patch antenna’s S11 plotted against frequency.

**Figure 9:**
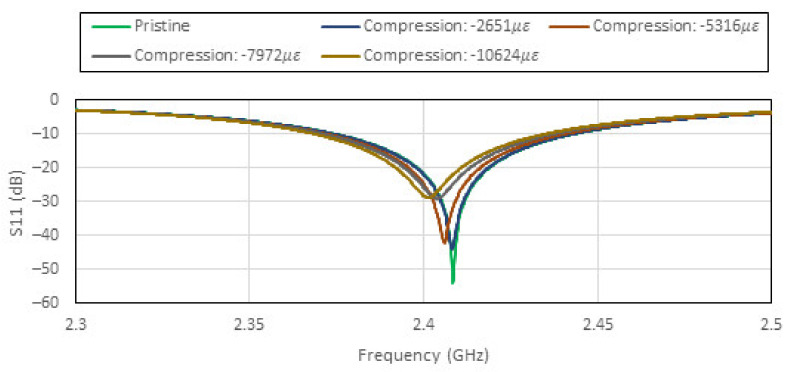
Effects of compressive loading on the patch antenna’s S11 plotted against frequency.

**Figure 10:**
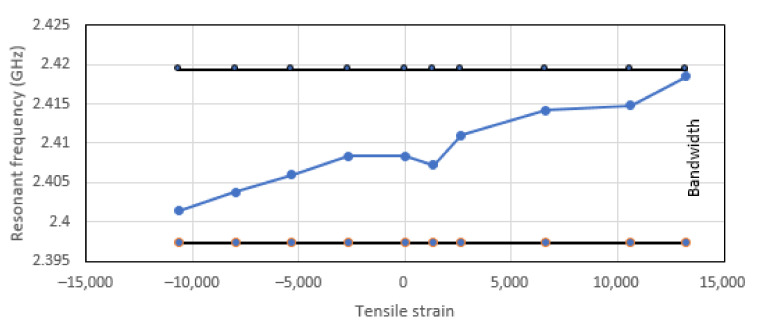
Resonant frequency excursions of the patch antenna which underwent tensile and compressive loading plotted against antenna bandwidth.

The uniaxial tensile and compressive loadings are applied in the X direction. Since the resonant frequency of the patch antenna is sensitive to the dimension b of the patch antenna (see Figure 3), the Poisson’s ratio induced effect is expected to be the dominant factor. Figure 8 and Figure 9 show the effects on the patch antenna resonance due to tensile and compressive loading. The bandwidth of the patch antenna as defined by IEEE802.11 is shown in Figure 10. These results suggest that the uniaxial loading that is applied predominantly in the X direction is unlikely to cause the CLAS to stray out of band.

**Figure 11:**
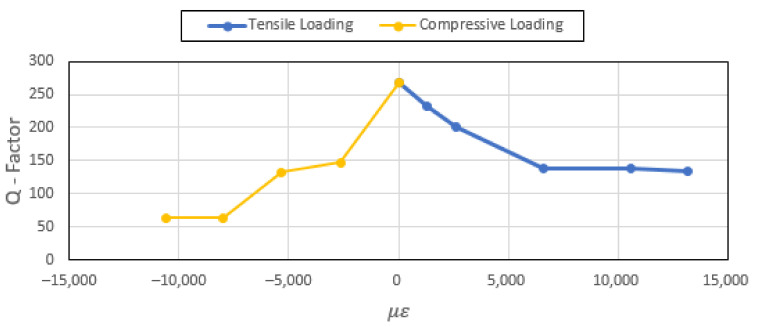
Effects of tensile and compressive loading on the patch antenna’s Q factor.

The Q factor of the antenna is significantly affected by the application of the mechanical loading (see Figure 11). An explanation for this effect are the changes in the dimensions $Z_{x}$ and $Z_{y}$ that arise from the deformation of the antenna due to mechanical loading. The effect on the Q factor is attributed to the changes in the dimensions of the recessed region thereby affecting the impedance matching conditions. These computational results will need to be verified experimentally since the Q factor of the CLAS is also dependant on the manufacturing tolerances. It is expected that small imperfections in the sharpness of the conductive elements in this matching region will manifest as significant changes in the Q factor.

### 3.3. Effects of Biaxial Loading

When applied to aircraft structures, it is likely that CLAS will be subjected to bi-axial state of stress. In this section, the two test cases reported include:

Case 1: X-direction tensile strain held approximately constant; with Y-direction tensile strain varied.

Case 2: Y-direction compressive strain held approximately constant; with X-direction tensile strain varied.

In Case 1, the b dimension of the antenna is directly affected by the mechanical loading, whilst in Case 2, the b dimension of the antenna is influenced by the Poisson’s ratio effect arising from the mechanical loading.

**Figure 12:**
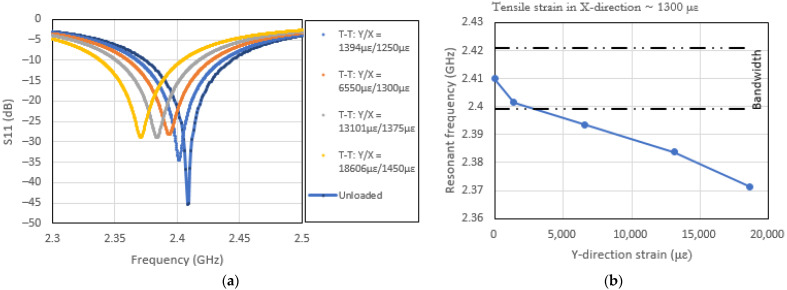
(**a**) Effects of bi-axial loading (tension–tension) on patch antenna’s S11 (Loading Case 1) and (**b**) resonant frequency excursion with respect to the CLAS bandwidth under bi-axial loading (tension–tension).

**Figure 13:**
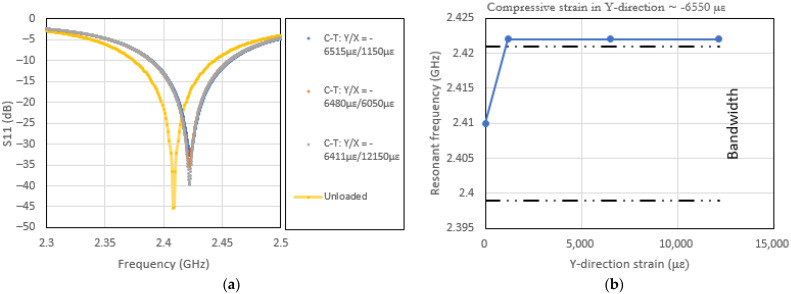
(**a**) Effects of bi-axial loading (compression–tension) on patch antenna’s S11 (Loading Case 2) and (**b**) resonant frequency excursion with respect to the CLAS bandwidth under bi-axial loading (compression–tension).

Figure 12a,b and Figure 13a,b show the effects of the stated bi-axial loading on the resonance of the CLAS and how they relate to the bandwidth of the patch antenna. The results in Figure 12a,b show the effects of predominant loading direction that aligns with the b dimension of the patch antenna. The results show the susceptibility of the CLAS to stray out of band under this loading configuration.

In Case 2, the resonant frequency is dominated by the loading applied in the Y direction. The varying X-direction loading will affect the b dimension via the Poisson’s ratio effect. In this respect, using the loading configuration as defined in Case 2, it is evident that the resonant frequency of the CLAS is relatively insensitive to the mechanical loading. However, the Q factor is sensitive to this loading configuration. This can be attributed to the fact that the Q factor is dependent on the geometric details in the recess region in the vicinity of the line-feed of the antenna. The geometry in the region will be affected by the X-direction loading.

### 3.4. Effects of Twist

**Figure 14:**
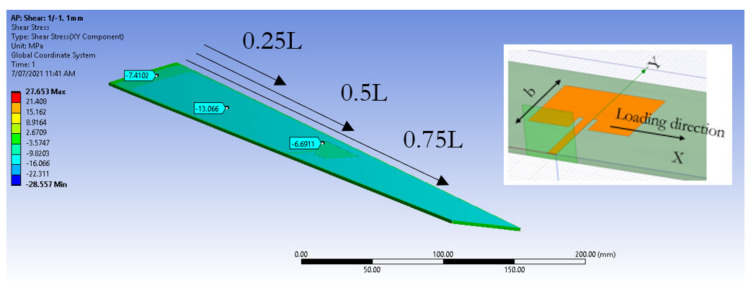
Deformed structure with embedded patch antenna under twist loading, with three positions indicating tested antenna locations.

Twist is a common loading configuration that aircraft wing structures are subjected to. Having documented the effects of mechanical loading on the frequency response of CLAS, this simulation will consider three cases where the CLAS will be located at 0.25 L, 0.5 L and 0.75 L from the fixed boundary of the specimen. The free end will be subjected to a torque of 3 N.m. Figure 14 shows the deformed structure with the applied loading.

**Figure 15:**
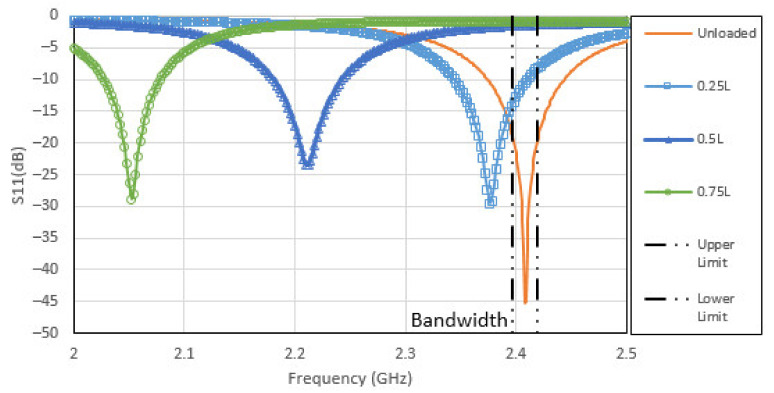
CLAS’s S11 plotted for the three antenna locations (0.25 L, 0.5 L, 0.75 L as shown in Figure 14) on the specimen experiencing twist loading.

The effects of this loading configuration on the CLAS’s S11 is shown in Figure 15, displaying the sensitivity of the resonant frequency to this form of loading. The potential of the CLAS straying out of band is shown in this result. These results underscore the importance of incorporating the mechanical loading that the structure is subjected to when designing and locating these CLAS.

### 3.5. Effects of Antenna Disbond

**Figure 16:**
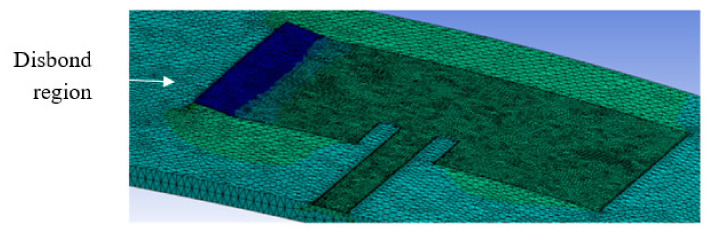
CLAS specimen loaded under tension, with the disbonded region elevated and coloured in dark blue.

This set of analyses reports on the presence of disbond in CLAS and the effect on its resonant behaviour. Disbond can arise due to manufacturing defects. One form of such manufacturing anomaly is a “kissing” bond where there is no structural connection between the adherends. The results below will describe the effects of the application of mechanical loading on a CLAS that is inflicted with this type of defect. The two disbond sizes considered are 5 mm and 10 mm from the edge of the CLAS (see Figure 16). The disbond is simulated by removing the connection between the disbonded region and the dielectric substrate. The model is then subjected to tensile loading only in the X direction.

**Figure 17:**
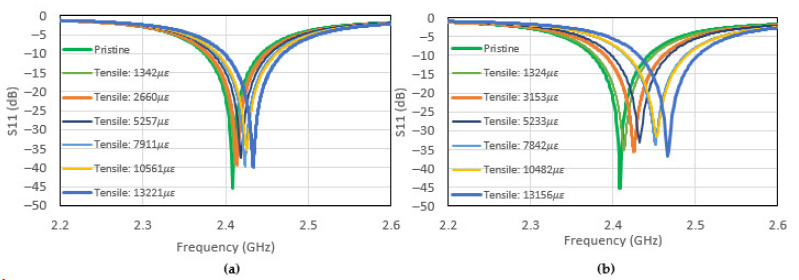
(**a**) CLAS’s S11 for the antenna with 5 mm disbond undergoing tensile loading at various levels and (**b**) CLAS’s S11 for the antenna with 10 mm disbond undergoing tensile loading at various levels.

The effects of the application of tensile loading on the S11 response are shown in Figure 17a,b. Given that the disbond will result in change of the a dimension of the CLAS (see Figure 3), the resonant behaviour can be explained by considering Equations (1)–(3), where the dimensions a and b are now affected by the manufacturing defect and the application of mechanical loading, respectively.

**Figure 18:**
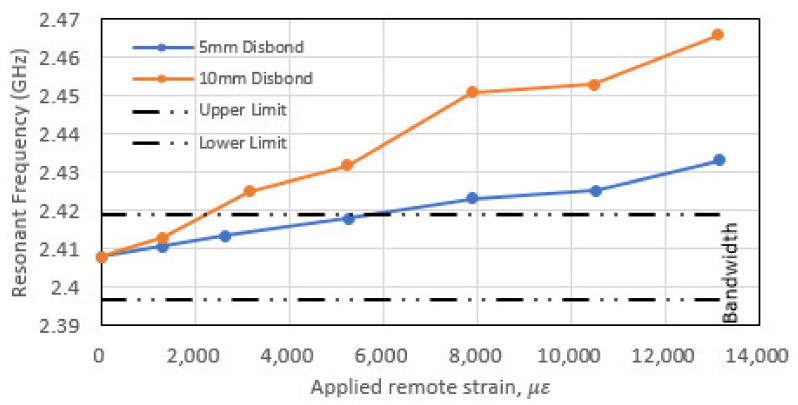
Resonance response of CLAS with disbond present (5 mm or 10 mm) with respect to mechanical loading.

The changes in the resonant frequency for each disbond case are plotted in Figure 18. These results show that the presence of a “kissing” disbond (that are difficult to identify using traditional inspection techniques) will not affect the resonance response of the CLAS prior to the application of any mechanical loading. However, the deformation of the disbond arising from mechanical loading will (a) change the relative permittivity in its vicinity and (b) bring into prominence the change in the a dimension in Equations (2) and (3). As shown in the results, the effects on the resonance of the CLAS straying out of band is amplified.

## 4. Discussion

The results reported herin demonstrate how sensitive the resonant behavior of the antenna is to deformations in its local geometry. It therefore follows that the resonant behaviour of patch antenna may stray out out of band when subjected to mechanical loading. Structural deformation of the antenna laminate result in a shift of the resonant frequency and a reduction in the antenna Q factor. Both effects will lead to degredation in comunications and potentially a complete loss of connectivity over WiFi. Deformation in the Y direction was shown to be responsible for the significant shifts in the resonant frequency (at ~2.4 GHz) through biaxial loading, with X-direction strain having no noteworthy effect.

To mitigate CLAS straying out of band or de-tuning will require an understanding of the loading configuration where the antenna is to be integrated. The effects of twisting further highlights the importance of location selection, with antenna closer to the site of loading experiencing more severe shifts to their resonant frequency compared to those further away. Furthermore, given that the metallised patch antenna is adhesively bonded to the di-electric substrate, the results show that the bond integrity may also the performance of the CLAS. For example, a “kissing” bond between the antenna element and the substrate introduces additional capacitance that de-tunes the antenna away from the desired operating point. Depending on the severity of the disbond the antenna will de-tune at different rates when the geometry experiences mechanical loading, with the larger disbonds encouraging more dramatic shifts in resonance frequency.

Experimental measurement of microwave performance under mechanical deformation is difficult because of the need for both a durable coaxial connection to the antenna that is well understood (from both a microwave and mechanical view) and will survive the mechanical loading, and a well-controlled microwave anechoic enclosure surrouding the mechanical loading frame. Previous work in [31] encountered difficulty ensuring that the SMA feed did not crack off before the microstrip failed. A small region was milled on the back face to expose the ground plane which was then filled with 502 epoxy, likely biasing the mechical results. The microstrip was characterised so the electric field was well contained, mitigating the need for an anechoic chamber. However, the patch was radiating meaning the resonance and Q factor will be inadvertently influenced by any surrounding reflections. Therefore, the numerical work presented above will set the scene and assist with the interpretation of the future experimental results. These numerical findings will be used to verify and delineate the true effects of mechanical loading on the RF response of the patch antenna.

The numerical results presented will be used in the design of a full-scale test article of a UAV wing structure. Detailed finite element analysis of the wing structure will be used to guide the design and subsequent integration of the WiFi communications antenna. To validate the design, mechanical testing of the wing structure will be conducted while simultaneousy monitoring the antenna performance.

## 5. Conclusions

This paper presented results on how tensile loading, biaxial loading, twisting, and antenna patch disbond influence the resonant frequency of a veil patch antenna. All cases were simulated using ANSYS HFSS, with both the RF response and quality factor analyzed. The results highlighted the effects of the various configurations of mechanical loading on the resonant behaviour of the patch antenna. The results also show how the RF response of the patch antenna is also affected by patch disbond. The outcomes of this work will help in progressing with planned experiments to measure the mechanical and electromagnetic properties of a veil embedded CLAS. The ultimate aim of this work is to improve future design and placement of patch antennas on a load-bearing deformable surface.

## Figures and Tables

**Table 1:** Mechanical properties for gms ep-280 s-glass.

$E_{x}\;(\mathrm{MPa})$	$E_{y}\;(\mathrm{MPa})$	$E_{z}\;(\mathrm{MPa})$	$\gamma_{xy}$	$\gamma_{yz}$	$\gamma_{xz}$	$G_{xy}\;(\mathrm{MPa})$	$G_{yz}\;(\mathrm{MPa})$	$G_{xx}\;(\mathrm{MPa})$
17,600	17,600	3500	0.33	0.33	0.33	6631	1250	1250

**Table 2:** Mechanical properties for carbon veil.

E (MPa)	γ	Bulk Modulus (MPa)	G (MPa)
8500	0.326	814	321

**Table 3:** Electromagnetic properties for gms ep-280 s-glass and carbon veil at 2.4 ghz.

Carbon Veil	GMS EP-280 S-Glass		
Bulk conductivity (S/m)	$\varepsilon_{r}$	$\mu_{r}$	Dielectric loss tangent
5333	4.37	1.00	0.02

## Data Availability

The raw/processed data required to reproduce these findings cannot be shared at this time as the data also forms part of an ongoing study.
